# Determinants of anxiety and depression among university teachers during third wave of COVID-19

**DOI:** 10.1186/s12888-023-04733-9

**Published:** 2023-04-07

**Authors:** Hamid Saeed, Amna Fakhar Qureshi, Muhammad Fawad Rasool, Muhammad Islam, Furqan Khurshid Hashmi, Amna Saeed, Rimsha Asad, Arfa Arshad, Azba Abid Qureshi

**Affiliations:** 1 Department of Pharmaceutics, College of Pharmacy, University of the Punjab, Allama Iqbal Campus, Lahore, 54000 Pakistan; 2 Department of Pharmacy Practice, Faculty of Pharmacy, Bahauddin Zakariya University, Multan, Pakistan; 3 Department of Pharmaceutical Sciences, Pak-Austria Fachhochschule: Institute of Applied Sciences and Technology, Mang Haripur, Pakistan

**Keywords:** Professors, Pakistan, DASS-21, Anxiety, Depression, COVID-19

## Abstract

**Background:**

To estimate the determinants of anxiety and depression among university teachers in Lahore, Pakistan, during COVID-19.

**Methods:**

A cross-sectional study was conducted by enrolling 668 teachers from the universities of Lahore, Pakistan. Data were collected using a questionnaire. Chi-square for significance and logistic regression for the association were used.

**Results:**

Majorly, the university teachers, with an average age of 35.29 years, had regular jobs (72.8%), job experience of > 6 years (51.2%) and good self-reported health (55.4%). The majority of the teachers were working as lecturers (59.6%), lecturing in arts (33.5%) or general science (42.5%) departments, having MPhil (37.9%) or master (28.9%) degrees, and teaching via synchronous video (59.3%) mode. Anxiety and depression, severe and extremely severe, were higher among lecturers, MPhil or master degree holders, teachers lecturing arts and general science subjects, and in those on contract employment. Anxiety was significantly associated with academic departments; arts (OR;2.5, *p* = 0.001) and general science (OR;2.9, *p* = 0.001), poor health status (OR;4.4, *p* = 0.018), and contractual employment (OR;1.8, *p* = 0.003). Depression was associated with academic departments; arts (OR;2.7, *p* = 0.001) and general science (OR;2.5, *p* = 0.001), and health status (OR;2.3, *p* = 0.001).

**Conclusion:**

Among university teachers, anxiety and depression, severe and extremely severe, were prevalent among lecturers having MPhil or master degrees, belonging to arts and general science departments, and among contract employees. Anxiety and depression were significantly associated with academic disciplines, lower cadre, and poor health status.

**Supplementary Information:**

The online version contains supplementary material available at 10.1186/s12888-023-04733-9.

## Background

The outbreak of COVID-19 was first reported in late December 2019 in the city of Wuhan, China and later spread to 209 countries, including Pakistan [1]. In Pakistan, the first case of COVID-19 was confirmed in Karachi, Sindh by the ministry of health, Government of Pakistan on February 26, 2020 [2]. The World Health Organization (WHO) declared it a public health emergency on January 30, 2020 [3]. To limit the spread of the contagion, strong measures of social distancing and lockdowns were imposed which led to a significant impact on social relations, and these restrictions had social, psychological, and educational consequences [4]. One of the most widely used measures to maintain social distancing and to decrease the spread of infection was the closure of educational institutions on a global scale [5], suspending face-to-face classes, and replacing them with virtual mode [6].

In Pakistan, during 3rd wave of the COVID-19 pandemic, as per the government reports (www.covid.gov.pk), almost 1,024,737 tests were performed with 672,391 (66%) confirmed cases of COVID-19 and more than 14,530 (2%) deaths, while the recovery stands at 90% (605,274). During this period the closing of the educational institutes with poor adaptability to digital mode of learning along with the lack of systematic and nationwide organized infrastructure of online teaching resulted in multifaceted stressful challenges for the policymakers, teachers, and students [7]. In this context, the Higher Education Commission of Pakistan (HEC) formed a COVID-19 Technology Support Committee to assist universities in developing distance learning guidelines or a strategic plan [8]. However, recent studies from Pakistan demonstrated a lack of preparedness, academic insufficiency, teacher dissatisfaction particularly for those involved in practical training, and more importantly internet connectivity and power shortage (electric load shedding) as the major challenges during this 3rd wave of the COVID-19 pandemic [7]. In Pakistan, the commonly used platforms for online education were Google Meet, Zoom, Learning Management System, and YouTube, yet with several issues, such as lack of privacy, access to technology, financial and technical issues and poor family support [8, 9].

Thus, during the COVID-19 pandemic times, with so many challenges, the emergent switch to a completely online mode has impacted teachers’ mental health, as many were not prepared for this instant change without adequate training on digital resources and lack of equipment for remote teaching [10]. Also identified by UNESCO and reported earlier, teachers demonstrate considerable anxiety, stress, and confusion due to the virtual learning mode during COVID-19 [11]. During the lockdowns, teachers suffered stress from having to adapt (in record time) to online lecturing (Besser et al., 2020). During the pandemic, a study conducted in three cities in China assessed the prevalence of anxiety among teachers, which was found to be 13.67%, with women being more anxious than men [12]. Another study from Spain reported that teachers experienced exhaustion, anxiety, and stress, with a higher propensity among women in comparison to men [13].

In Pakistan, the situation after the disruption of the education system became very bleak – possibly due to a lack of training, resources, and technological equipment. Very little literature evidence exists concerning the evaluation of the mental health of university teachers in Pakistan facing enormous challenges during the COVID-19 pandemic. One study from Pakistan describes that teachers were confronted with multiple fronts during the pandemic, such as COVID-19 infection, limited recourses, and digital transformation all affecting their psychological state, which resulted in psychological distress and poor job satisfaction [14]. However, the psycho-emotional concerns and determinants of anxiety and depression among university teachers were not touched upon. Thus, we aimed to estimate the determinants of anxiety and depression among university teachers during the COVID-19 pandemic in Lahore, Pakistan.

## Method

### Ethical consideration

The study was approved by the Human Research Ethics Committee, University of the Punjab, reference #;003/21/UCP. Informed consent was obtained from the participants before distributing self-administered questionnaires. All the participants were briefed about the nature and objective of the research.

### Study Design

A descriptive cross-sectional study was conducted by enrolling university teachers, from different universities across Lahore, Pakistan – Punjab University (PU), Lahore College for Women University (LCWU), Kinnaird College for Women (KCW), Government College University (GCU), Bahauddin Zakariya University (BZU) and University of Central Punjab (UCP). Data were collected for a period of 6 months, from December 2020 – May 2021, amid 3rd wave of the COVID-19 pandemic, by field administrators via questionnaires. Data were segregated based on gender, males (n = 283) and females (n = 385). The questionnaire was divided into three sections, i-e., basic demographics, the preventive measures during COVID-19, and the assessment of Anxiety and Depression using the Depression Anxiety and Stress Scale (DASS-42), only 28 items multiple choice questions, about anxiety and depression were administered to estimate the scores of anxiety and depression.

### Participants

A total of 1000 questionnaires were distributed among university teachers − 220 questionnaires were partially filled and 112 questionnaires were not returned to the field administrators. Thus, only 668 teachers were enrolled in the study, 283 were male and 385 were female teachers. The sampling frame consisted of university Lecturers, Assistant Professors, Associate Professors, and Professors. The enrollments were settled as per the study inclusion and exclusion criteria.

#### Inclusion criteria

All the teachers of various departments in public/private universities of Pakistan, irrespective of age, gender, ethnicity, religion, social class, and willingness to participate were enrolled in the study.

#### Exclusion criteria

All the school or retired teachers and those not willing to participate were excluded from the study.

### Data collection

Data were collected utilizing a comprehensive instrument of measure designed after an extensive literature review [15–18] (supplementary material S1). The self-administered questionnaire was sent to subject experts/academicians for content validation, thereafter their expert opinion was incorporated to make the questionnaire simpler and more objective driven. The reliability of the questionnaire was evaluated with Cronbach’s alpha (0.78) using SPSS version 22. Face validation of the questionnaire was done by conducting a pilot study on 20 teachers and the feedback was incorporated into the final questionnaire. The questionnaires were disseminated among teachers of Punjab University (PU), Lahore College for Women University (LCWU), Kinnaird College for Women (KCW), Government College University (GCU), Bahauddin Zakariya University (BZU), and University of Central Punjab (UCP) during physical teaching mode. The questionnaire was outlined in 2 sections:

#### Section 1

consisted of three portions; Basic demographics such as age, gender, employment status, and marital status; Teacher’s academic profiles, such as teaching mode, job experience, Health, and psycho-emotional profiles; self-rating health status, attention, worried, fear, and motivation levels. Attention, worried, fear, and motivation levels were rated on a scale of 1–10, where 8–10 was rated as high, 5–7 as moderate and < 5 as low.

#### Section 2

contains questions about health status followed by questions on anxiety and depression, later assessed based on the Severity-rating index.

#### Study instrument

The score for each respondent over each sub-scale was evaluated by the Severity-rating index (DAAS) [19]. DASS-42 items questionnaire, a previously validated and standardized survey instrument, is a set of three self-reported scales designed to estimate the clinically significant negative emotional states of stress, anxiety, and depression and has been utilized in the Pakistani population before [16]. The depression scale assesses devaluation of life, dysphoria, lack of involvement, anhedonia, and inertia, and the anxiety scale assesses skeletal muscle effects, situational anxiety, shaky and panicky behavior. DASS questionnaire severity rating index was used to define levels of anxiety (normal; 0–7, mild; 8–9, moderate; 10–14, severe; 15–19 and extremely severe; +20) and depression (normal; 0–9, mild; 10–13, moderate; 14–20, severe; 21–27 and extremely severe; +28).

### Data Analysis

The data were analyzed using SPSS (IBM, version 22) unless otherwise reported. Descriptive analysis was performed to estimate the percentages and frequencies of the categorical variables. Associations of dependent variables including demographics, practices during COVID-19, precautions and preventive measures, and independent variables such as gender, anxiety scale, and depression scale were estimated using Pearson’s Chi-square. Odds ratios were estimated using binary logistic regression. An alpha value of 0.05 or less was considered statistically significant.

## Results

### Demographic distribution of University Teachers

Population demographics are summarized in Table 1. Data revealed that the mean age of male and female teachers was 32.07 ± 7.39 and 39.66 ± 9.36 years, respectively. The majority of the enrolled teachers were females (57.6%), married (70.2%), had regular employment status (72.8%), had good self-reported health (55.4%), and had teaching job experience of more than 6 years (51.2%). Besides, almost 59.1% of teachers worked ≤ 6 h a day during the COVID-19 pandemic with most COVID-19 cases either in their family (34.2%) or among their friends (23.8%) (Table 1).

**Table 1:** General Demographics of University Teachers

Characteristics		Mean	Standard Deviation
**Age in years**	*Cumulative*	35.29	9.1
	*Male*	32.1	7.4
	*Female*	39.7	9.4
		**Frequency (** ***n*** **)**	**Percentage** ***(%)***
**Marital Status**	*Married*	469	70.2
	*Unmarried*	199	29.8
**Gender**	*Male*	283	42.4
	*Female*	385	57.6
**Employment Status**	*Regular*	486	72.8
	*Part time*	33	4.9
	*Contract*	149	22.3
**Job Experience**	*≤ 6 (years)*	326	48.8
	*> 6 (years)*	342	51.2
**Avg Office Hours**	*≤ 6(hours)*	395	59.1
	*> 6(hours)*	273	40.9
**Self-reported Health Status**	*Good*	370	55.4
	*Fair*	286	42.8
	*Poor*	12	1.8
**Anyone Effected with COVID-19**	*Family*	229	34.2
	*Friends*	159	23.8
	*Colleagues*	141	21.1
	*Students*	72	10.8
	*No One*	67	10

### Academic and psycho-emotional profiles of University Teachers

Teachers’ academic and psycho-emotional profiles are summarized in Table 2. Data revealed that the majority of the teachers had MPhil (37.9%) as their terminal degree followed by the master (28.9%) and Ph.D. (26.5%). Out of a total, 42.5% were working in the general science department followed by arts (33.5%) and medical sciences (18.3%). Among the enrolled population, almost 60% of teachers were working as lecturers, 16.2% as assistant professors and only 7.9% as professors. During the COVID-19 pandemic, the majority of the teachers used synchronous videos (59.3%) as a mode of teaching followed by social media (16.9%) and institutional management system (15.6%). When it comes to psycho-emotional levels, almost more than 58% of teachers had moderate levels of attention, motivation, worried and fear during COVID-19 classes (Table 2).

**Table 2:** Academic and Psycho-emotional Profiles of University Teacher’s

Characteristics		Frequency (*n*)	Percentage *(%)*
**Academic Degrees**	*PhD*	177	26.5
	*MPhil*	253	37.9
	*Masters*	193	28.9
	*Bachelors*	45	6.7
**Academic Departments**	*Arts*	224	33.5
	*Social Sciences*	38	5.7
	*General Sciences*	284	42.5
	*Medical Sciences*	122	18.3
**Academic Designations**	*Professor*	53	7.9
	*Associate Professor*	43	6.4
	*Assistant Professor*	108	16.2
	*Lecturer*	464	59.6
**Mode of teaching during COVID-19**	*Social media*	113	16.9
	*Institutional management*	104	15.6
	*Synchronous videos*	396	59.3
	*Recorded lecture*	34	5.1
	*Others*	21	3.1
**Attention levels**	*High*	201	30
	*Moderate*	403	60.3
	*Low*	64	9.7
**Motivation levels**	*High*	194	29
	*Moderate*	394	58.9
	*Low*	80	11.9
**Worried levels**	*High*	143	21.4
	*Moderate*	403	60.3
	*Low*	118	17.7
**Fear levels**	*High*	90	13.5
	*Moderate*	419	62.7
	*Low*	159	23.8

### Anxiety and depression levels among University Teachers during COVID-19 based on academic profiles

The distribution of anxiety and depression among university teachers during COVID-19 based on teaching profiles are summarized in Table 3. As shown in Table 3, significant differences were observed with regard to the frequency distribution in the levels of anxiety and depression in academic profiles of the teachers, such as academic departments, academic designations, academic employment status, and academic degrees. Data suggested that among the academic departments, the majority of the teachers of arts (*Anxiety*: **S;** 35.4%, **ES;** 32.6%, *p* = 0.0001, *Depression*: **S;** 29%, **ES;** 60%, *p* = 0.0001) and general sciences (*Anxiety*: **S;** 49.2, **ES;** 57.3, *p* = 0.0001, *Depression*: **S;** 56.5%, **ES;** 40%, *p* = 0.0001) departments were having severe (**S**) and extremely sever (**ES**) levels of anxiety and depression. Additionally, anxiety and depression, severe and extremely server were more frequent among lecturers (*Anxiety*: **S;** 89.2%, **ES;** 89.9%, *p* = 0.0001, *Depression*: **S;** 95.7%, **ES;** 86.7%, *p* = 0.0001), teachers with MPhil degrees (*Anxiety*: **S;** 63.1%, **ES;** 55.1%, *p* = 0.0001, *Depression*: **S;** 65.2%, **ES;** 26.7%, *p* = 0.0001) and those with regular (*Anxiety*: **S;** 52.3%, **ES;** 51.7%, *p* = 0.0001, *Depression*: **S;** 55.1%, **ES;** 73.3%, *p* = 0.0001) and contract employment (*Anxiety*: **S;** 36.9%, **ES;** 39.3%, *p* = 0.0001, *Depression*: **S;** 33.3%, **ES;** 13.3%, *p* = 0.0001) status (Table 3).

**Table 3:** Distribution of Anxiety and Depression Levels among University Teachers based on Academic profiles

Characteristics	Anxiety Levels, *n* (*%*)					
	*Normal*	*Mild*	*Moderate*	*Severe*	*Extremely severe*	*P-values*
**Academic Departments**						
*Arts* *Social sciences* *General sciences* *Medical sciences*	127 (31.9) 31 (7.8) 149 (37.4) 91 (22.9)	19 (45.2) 2 (4.8) 12 (28.6) 9 (21.4)	26 (36.1) 2 (2.8) 38 (52.8) 6 (8.3)	23 (35.4) 2 (3.1) 32 (49.2) 8 (12.3)	29 (32.6) 1 (1.1) 51 (57.3) 8 (9)	0.0001**
**Academic Designations**						
*Professor* *Associate professor* *Assistant Professor* *Lecturer*	35 (8.8) 35 (8.8) 78 (19.6) 250 (62.8)	5 (11.9) 4 (9.5) 12 (28.6) 21 (50)	10 (13.9) 0 (0) 7 (9.7) 55 (76.6)	1 (1.5) 2 (3.1) 4 (6.2) 58 (89.2)	0 (0) 2 (2.2) 7 (7.2) 80 (89.9)	0.0001**
**Academic Degrees**						
*PhD* *MPhil* *Masters* *Bachelors*	137 (32.2) 128 (34.4) 107 (26.9) 26 (6.5)	18 (42.9) 13 (31) 8 (19) 3 (7.1)	15 (20.8) 13 (18.1) 35 (48.6) 9 (12.5)	8 (12.3) 41 (63.1) 15 (23.1) 1 (1.5)	6 (6.7) 49 (55.1) 28 (31.5) 6 (6.7)	0.0001**
**Employment status**						
*Regular* *Part time* *Contract*	317 (79.6) 14 (3.5) 67 (16.8)	33 (78.6) 3 (7.1) 6 (14.3)	54 (75) 1 (1.4) 17 (23.6)	34 (52.3) 7 (10.8) 24 (36.9)	46 (51.7) 8 (9) 35 (39.3)	0.0001**
	**Depression Levels**, ***n*(***%***)**					
	***Normal***	***Mild***	***Moderate***	***Severe***	***Extremely severe***	***P-values***
**Academic Departments**						
*Arts* *Social sciences* *General sciences* *Medical sciences*	130 (32.4) 23 (5.7) 154 (38.4) 94 (23.4)	33 (37.5) 10 (11.4) 38 (43.2) 7 (6)	32 (33.7) 4 (4.2) 47 (49.5) 12 (12.6)	20 (29) 1 (1.4) 39 (56.5) 9 (13)	9 (60) 0 (0) 6 (40) 0 (0)	0.001**
**Academic Designations**						
*Professor* *Associate professor* *Assistant Professor* *Lecturer*	47 (11.7) 33 (8.2) 80 (20) 241 (60.1)	3 (3.4) 6 (6.8) 16 (18.2) 36 (71.6)	2 (2.1) 2 (2.1) 10 (10.5) 81 (85.3)	0 (0) 2 (2.9) 1 (1.4) 66 (95.7)	1 (6.7) 0 (0) 1 (6.7) 13 (86.7)	0.0001**
**Academic Degrees**						
*PhD* *MPhil* *Masters* *Bachelors*	141 (35.2) 128 (31.9) 107 (26.7) 25 (6.2)	23 (26.1) 29 (33) 29 (33) 7 (8)	4 (4.2) 47 (49.5) 34 (35.8) 10 (10.5)	7 (10.1) 45 (65.2) 14 (20.3) 3 (4.3)	2 (13.3) 4 (26.7) 9 (60) 0 (0)	0.0001**
**Employment status**						
*Regular* *Part time* *Contract*	316 (78.8) 14 (3.5) 71 (17.7)	67 (76.1) 3 (3.4) 18 (20.5)	54 (56.8) 6 (6.3) 35 (36.8)	38 (55.1) 8 (11.6) 23 (33.3)	11 (73.3) 2 (13.3) 2 (13.3)	0.0001**

### Association of Teacher’s characteristics with anxiety and depression

The association of teachers’ academic profiles, such as gender, employment status, academic degree, academic departments, self-reported health, and academic designations with anxiety and depression is summarized in Tables 4 and 5. Data suggested that anxiety was strongly associated with the academic departments; with a higher likelihood of anxiety among teachers lecturing arts (OR; 2.5, *p* = 0.001) and general science (OR; 2.9, *p* = 0.001) subjects, with self-reported health; higher odds of anxiety in teachers with fair (OR; 2.3, *p* = 0.001) and poor (OR; 4.4, *p* = 0.018) health status, and with employment status; teachers on contract (OR; 1.8, *p* = 0.003) were more likely to have anxiety (Table 4).

**Table 4:** Association of Teacher’s characteristics with Anxiety

Characteristics	Anxiety, *n* = 668		Binary Logistic Regression	*p*-values
	YES, *n* = 270 (*%*) (*Score: 8–20+*)	NO, *n* = 398 (*%*) (*Score: 0–7*)	*OR (95% Cl)*	
**Gender**				
Male Female	108 (40) 162 (60)	175 (44) 223 (56)	1.1 (0.72–1.5) (*reference*)	0.839
**Academic Designation**				
Professor Associate Professor Assistant Professor Lecturer	18 (6.7) 8 (3) 30 (11.1) 214 (79.3)	35 (8.8) 35 (8.8) 78 (19.6) 250 (62.8)	(*reference*) 1.4 (0.64–3.1) 0.84 (0.38–1.8) 0.59 (0.21–1.7)	0.401 0.643 0.315
**Terminal Degree**				
PhD MPhil Masters Bachelor	49 (18.1) 116 (43) 86 (31.9) 19 (7)	128 (32.2) 137 (34.4) 107 (26.9) 26 (6.5)	(*reference*) 1 (0.56–1.9) 1 (0.56–1.9) 0.8 (0.34–1.9)	0.933 0.914 0.609
**Academic Department**				
Arts Social Sciences General Science Medical Science	97 (35.9) 7 (2.6) 135 (50) 31 (11.5)	127 (31.9) 31 (7.8) 149 (37.4) 91 (22.9)	2.2 (1.3–3.8) 0.60 (0.23–1.58) 2.78 (1.64–4.69) (*reference*)	0.004* 0.302 0.0001**
**Self-reported Health Status**				
Good Fair Poor	117 (43.3) 145 (53.7) 8 (3)	253 (63.6) 141 (35.4) 4 (1)	(*reference*) 2.46 (1.74–3.5) 3.86 (1–14.8)	0.0001** 0.05*
**Employment Status**				
Regular Part time Contract	169 (62.6) 19 (7) 82 (30.4)	317 (79.6) 14 (3.5) 67 (16.8)	(*reference*) 1.63 (0.74–3.62) 2.1 (1.34–3)	0.225 0.001**

p-values; 0.05–0.002*, ≤ 0.001**

When it comes to depression, males were more likely to experience depression (OR; 1.5, *p* = 0.023). As observed with anxiety, depression was significantly associated with academic departments, a higher propensity of depression among teachers lecturing arts (OR; 2.7, *p* = 0.001), general sciences (OR; 2.7, *p* = 0.017), and even social science (OR; 2.5, *p* = 0.001). Besides, higher chances of depression among teachers with fair (OR; 2.3, *p* = 0.001) and poor (OR; 2.5, *p* = 0.122) health status (Table 5). Besides, depression was significantly associated with academic designation; associate professors (OR; 2.6, *p* = 0.05), though insignificant yet higher likely hood among assistant professors (OR; 2.1, *p* = 0.153) and lecturers (OR; 1.8, *p* = 0.335). Although not significant, the chances of depression were higher among teachers having bachelor’s degrees (OR; 1.6, *p* = 0.070) and those working part-time (OR; 2, *p* = 0.062) (Table 5).

**Table 5:** Association of Teacher’s characteristics with Depression

Characteristics	Depression, *n* = 668		Binary Logistic Regression	*p*-values
	YES, *n* = 251 (*%*) (*Score: 10–28+*)	NO, *n* = 417 (*%*) (*Score: 0–9*)	*OR (95% Cl)*	
**Gender**				
Male Female	98 (39) 153 (61)	185 (44.4) 232 (55.6)	1.13 (0.78–1.64) (*reference*)	0.513
**Academic Designation**				
Professor Associate Professor Assistant Professor Lecturer	6 (2.4) 9 (3.6) 28 (11.2) 208 (82.9)	47 (11.3) 34 (8.2) 80 (19.2) 256 (61.4)	(*reference*) 3.52 (1.1–11.73) 2.71 (1–7.34) 4.32 (1.59–11.7)	0.04* 0.05* 0.004*
**Terminal Degree**				
PhD MPhil Masters Bachelor	35 (13.9) 116 (46.2) 81 (32.3) 19 (7.6)	142 (34.1) 137 (32.9) 112 (26.9) 26 (6.2)	(*reference*) 1.53 (0.64–3.64) 1.68 (0.88–3.18) 1.83 (0.98–3.41)	0.339 0.114 0.052
**Academic Department**				
Arts Social Sciences General Science Medical Science	93 (37.1) 15 (6) 119 (47.4) 24 (9.6)	131 (31.4) 23 (5.5) 165 (39.6) 98 (23.5)	2.52 (1.43–4.47) 2.92 (1.24–6.86) 3.16 (1.8–5.56) (*reference*)	0.002* 0.014* 0.0001**
**Self-reported Health Status**				
Good Fair Poor	109 (43.4) 136 (54.2) 6 (2.4)	261 (62.6) 150 (36) 6 (1.4)	(*reference*) 2.29 (1.62–3.24) 1.72 (0.47–6.3)	0.0001** 0.414
**Employment Status**				
Regular Part time Contract	163 (64.9) 18 (7.2) 70 (27.9)	332 (77.5) 15 (3.6) 79 (18.9)	(*reference*) 1.81 (0.81–4.1) 1.4 (0.91–2)	0.146 0.129

p-values; 0.05–0.002*, ≤ 0.001**

## Discussion

Needless to say, COVID-19 has affected people from all walks of life, yet the mental state of the teachers is rarely touched upon. Several literature reports suggested that teachers suffered stress from having to adapt to the virtual mode of teaching, which resulted in symptoms of anxiety, depression, and often sleep disturbances among teachers [20]. In the present study, data revealed that the majority of the teachers were regular employees, with job experience of > 6 years and good self-reported health. Besides, more than 60% of teachers hold MPhil and master degrees, > 59% of teachers were lecturers, using a synchronous mode of teaching, and demonstrated moderate levels of attention, motivation, worried and fear during the COVID-19 pandemic. The levels of anxiety and depression were significantly different among teachers based on academic profiles. The chances of anxiety were significantly higher in teachers lecturing in arts and general sciences, those having poor self-reported health and on contracts. The probability of depression was significantly higher among teachers lecturing arts, general sciences, and social sciences subjects, working as lecturers, associate professors, and with poor self-reported health.

During COVID-19, the synchronous video (> 58%) was the most frequent mode of teaching followed by the intuitional management system, corroborating previous reports from Taiwan and Saudi Arabia (SA), where almost 40% of teachers used synchronous teaching mode [21] and the majority of the medical students in SA were receptive to synchronous teaching mode [22]. Teaching is considered a highly stressful profession because on daily basis teachers have to cope with emotional stress, conflict mediation, and work overload [23]. In addition to the psychological impact of social isolation, teachers, during the pandemic and in a short period, have to adapt to remote teaching and conform to their personal and job-related responsibilities besides fear of health and job security – all mounting towards tremendous mental and emotional stress [23]. In this context, a recent meta-analysis regarding the mental and emotional impact of COVID-19 on teachers showed that 17% experience anxiety, 19% depression, and 33% stress [18]. This study estimated the levels of anxiety and depression among teachers based on academic profiles and concluded that anxiety and depression, severe and extremely severe, were more prevalent among teachers of arts and general sciences departments, lecturers, MPhil or master degree holders and among contract employees. The possible reasons among arts and general science teachers may be linked to the incomprehensive understanding and ambiguity regarding COVID-19 and its preventive measures. In lecturers and teachers with MPhil or master degrees, this may be linked to a higher workload on being junior, not only their own but also of senior faculty members, involvement in research, either Ph.D. research or others, and risk of contagion due to greater need for communicating with the students as well as the possibility of greater contact with the professionals and university authorities – corroborating previous reports from Bangladesh [24] and China [25]. Anxiety and depression among contract employees may be linked to job insecurity in pandemic times due to the closure of face-to-face classes and the need to cut short the academic syllabus. In this regard, as reported previously, teachers with work stability of a few months and fixed contracts experienced frequent anxiety compared to teachers with indefinite contracts [17, 20]. Teachers with self-reporting health status as fair and poor were more likely to develop anxiety and depression due to sentient feelings of an already existing health problem, corroborating previous findings that teachers who were already ill or living with ill/COVID-19 patients had higher anxiety and depression [15, 26].

Seemingly, this emergency e-learning provoked diverse changes in teacher’s workload, such as challenges in task identity, the significance of the assigned task, diversity of the digital skills, autonomy, feedback, and social issues – all due to an abrupt and unplanned change in learning context not chosen by the teachers. Nonetheless, lower cadre, having MPhil or master degrees, instability in the job, poor health status, or having ill/COVID-19 patients at home and in the non-medical academic field were associated factors contributing to anxiety and depression among university teachers.

### Practical implications

During the COVID-19 pandemic, transferring teaching content to the online mode and still making its relevance is a great challenge for teachers at all academic levels. An emergent switch to e-learning without one’s consent and preparation can provoke changes in teachers’ workload and motivation toward their assigned academic tasks. Therefore, it is necessary to develop and initiate policies and interventions to protect teachers’ mental health considering that their decent mental health status may predict students’ psycho-emotional well-being and commitment to studies.

Thus, any forthcoming intervention or policy must integrate junior teachers, those belonging to arts or social/general sciences departments, on contract employment, and having poor health status to strengthen teachers’ mental health, probably by providing pedagogical and psycho-emotional support.

### Study Limitations

The cross-sectional design of the study does not allow to document responses of the respondents that may change over a period of time. To our knowledge, due to the non-availability of literature reports from Pakistan, a direct comparison cannot be made. The non-probability sampling may introduce selection bias as participation was voluntary.

## Conclusions

Data suggested that the majority of the teachers belonged to arts and general science academics with MPhil as their terminal degrees and worked in the lower cadre, i-e., lecturer, employing synchronous videos as a mode of teaching. Anxiety and depression, severe and extremely severe, were higher among lecturers holding MPhil or master degrees, lecturing in arts or general sciences subjects, and teaching either part-time or as contract employees. Furthermore, anxiety and depression were significantly associated with teachers working on contract appointments, in lower cadres, and having poor health status. Thus, teachers need professional assistance to cope with their mental health issues in these pandemic times.

## Electronic supplementary material

Below is the link to the electronic supplementary material.

Supplementary Material 1 Questionnaire

## Data Availability

Data sets used or analyzed are available from the corresponding author on reasonable request.

## Abbreviations

COVID-19: Corona virus disease 2019
WHO: World Health Organization
UNESCO: United Nations Educational and Cultural Organizations
PU: Punjab University
LCWU: Lahore College for Women University
KCW: Kinnaird College Lahore
GCU: Government College Lahore
BZU: Bahauddin Zakariya University
UCP: University of Central Punjab
SOPs: Standard operating procedures
M: Male
F: Female
OR: Odds ratio
Avg: Average
SD: Standard deviation
PhD: Doctor of philosophy
MPhil: Master of philosophy
DASS-42: Depression Anxiety and Stress Scale
