# Supplementary material for: Determinants of anxiety and depression among university teachers during third wave of COVID-19
Source: BMC Psychiatry. 2023 Apr 7;23:233. doi: 10.1186/s12888-023-04733-9 (PMC10080511; doi:10.1186/s12888-023-04733-9)
Supplement: Supplementary file 1 — Supplementary Material 1 Questionnaire [file 12888_2023_4733_MOESM1_ESM.docx]

**Determinants of Anxiety and depression among university teachers during 3^rd^ Wave of COVID-19**

**Section – I (**Demographics, academic and pyscho-emotional profiles**)**

**Age:** ________ **Gender:** M F **Terminal degree:** ____________

**Marital status:** M F **Designation:** _________ **Department:** _________

**Employment status:** regular part time contract

**Anyone infected with corona:** family friends colleagues’ students

**Teaching mode during corona:** social media via institution management synchronous video

(zoom, google meet) asynchronous (recorded lectures) Others

**Attention, worried, fear and motivation Levels to be measured on a scale of 1 - 10**

**Attention level:** high (8-10) moderate (5-7) low (<5) **Worried level:** high moderate low

**Fear level:** high moderate low **Motivation level:** high moderate low

**Self-rated health status:** good fair poor **Job experience (***years***):** _____________

**Section – II (**Anxiety and depression**)**

**Depression Anxiety and Stress Scale (DAAS)**

Please read each statement and circle a number 0, 1, 2 or 3 which indicates how much the statement applied to you *over the past week*. There are no right or wrong answers.

Do not spend too much time on any statement.

*The rating scale is as follows:*

**0** Did not apply to me at all

**1** Applied to me to some degree, or some of the time

**2** Applied to me to a considerable degree, or a good part of time

**3** Applied to me very much, or most of the time

| **Sr. #** | **Questions** | **Scoring** | | | |
| --- | --- | --- | --- | --- | --- |
| 2 | I was aware of dryness of my mouth | 0 | 1 | 2 | 3 |
| 3 | I couldn't seem to experience any positive feeling at all | 0 | 1 | 2 | 3 |
| 4 | I experienced breathing difficulty (e.g, excessively rapid breathing, breathlessness in the absence of physical exertion) | 0 | 1 | 2 | 3 |
| 5 | I just couldn't seem to get going | 0 | 1 | 2 | 3 |
| 7 | I had a feeling of shakiness (eg, legs going to give way) | 0 | 1 | 2 | 3 |
| 9 | I found myself in situations that made me so anxious I was most relieved when they ended | 0 | 1 | 2 | 3 |
| 10 | I felt that I had nothing to look forward to | 0 | 1 | 2 | 3 |
| 13 | I felt sad and depressed | 0 | 1 | 2 | 3 |
| 15 | I had a feeling of faintness | 0 | 1 | 2 | 3 |
| 16 | I felt that I had lost interest in just about everything | 0 | 1 | 2 | 3 |
| 17 | I felt I wasn't worth much as a person | 0 | 1 | 2 | 3 |
| 19 | I perspired noticeably (eg, hands sweaty) in the absence of high temperatures or physical exertion | 0 | 1 | 2 | 3 |
| 20 | I felt scared without any good reason | 0 | 1 | 2 | 3 |
| 21 | I felt that life wasn't worthwhile | 0 | 1 | 2 | 3 |
| 23 | I had difficulty in swallowing | 0 | 1 | 2 | 3 |
| 24 | I couldn't seem to get any enjoyment out of the things I did | 0 | 1 | 2 | 3 |
| 25 | I was aware of the action of my heart in the absence of physical exertion (eg, sense of heart rate increase, heart missing a beat) | 0 | 1 | 2 | 3 |
| 26 | I felt down-hearted and blue | 0 | 1 | 2 | 3 |
| 28 | I felt I was close to panic | 0 | 1 | 2 | 3 |
| 30 | I feared that I would be "thrown" by some trivial  but unfamiliar task | 0 | 1 | 2 | 3 |
| 31 | I was unable to become enthusiastic about anything | 0 | 1 | 2 | 3 |
| 34 | I felt I was pretty worthless | 0 | 1 | 2 | 3 |
| 36 | I felt terrified | 0 | 1 | 2 | 3 |
| 37 | I could see nothing in the future to be hopeful about | 0 | 1 | 2 | 3 |
| 38 | I felt that life was meaningless | 0 | 1 | 2 | 3 |
| 40 | I was worried about situations in which I might panic and make a fool of myself | 0 | 1 | 2 | 3 |
| 41 | I experienced trembling (eg, in the hands) | 0 | 1 | 2 | 3 |
| 42 | I found it difficult to work up the initiative to do things | 0 | 1 | 2 | 3 |
| TOTAL |  |  |  |  |  |
